# Revisiting English as a foreign language teachers’ professional identity and commitment in social media-focused professional development

**DOI:** 10.3389/fpsyg.2022.992038

**Published:** 2022-09-15

**Authors:** Wenjiang Ping

**Affiliations:** School of Foreign languages, Xinxiang Medical University, Xinxiang, China

**Keywords:** professional development, social media focused, EFL education, commitment, teacher professional identity

## Abstract

These days, technology advancement has inspired a large number of educators to employ social media in the English as a foreign language (EFL) context. But, some EFL educators are yet unwilling to use such chances, because they are left untrained. Therefore, applying professional development (PD) in this field appears necessary as it is regarded as the main cause of improving educators’ education activity, and proposing new education approaches. To strengthen the academic investment in educators’ professional learning, comprehending elements affecting educators’ performance of learning from PD is vital. Lower commitment degrees were specified as an element that impedes EFL educators from employing social media in EFL education. Moreover, developing the professional identity (PI) of educators is an important issue that straightly pertains to educators’ performance, which assists educators both in comprehending their professional lives and presenting them with a framework to elucidate, expand and contemplate their professional activities. Stimulated by the interest in research on social media-focused courses, this review inspects how teachers’ commitment and identity affect EFL teachers’ implementation of social media in PD. In a nutshell, implications for stakeholders of the study are presented. Indeed, the social media as an impotent device should be incorporated in PD programs to develop both teachers’ commitment and identity. Faculty members should establish workshops, and seminars to arrange for a platform for teachers to use social media to support learning.

## Introduction

Discussing the pedagogical aspects of technology, van Braak (2001) asserts that the application of technology in educational contexts enhances learning, creating useful learning opportunities. These conditions make it easier for the students to learn regardless of time and place through social media which is a noteworthy technological facet that has become extensively employable in education (Kiedrowski et al., 2015). Dabbagh and Kitsantas (2012) define social media as a diversity of networked instruments or technologies that highlight the social facets of the Internet as a waterway for communication, cooperation, and resourceful appearance, and is often substitutable with the concept such as social software. EFL educators might utilize social media for teaching in various ways; for example, Hall et al. (2010) found that some educators utilized associative whiteboards to project material, while others utilized them to answer learners’ questions. Emphasizing the utilization of the Web, Trentin (2008) determined various utilization trends among EFL educators: some utilize it to offer class information and educational content, some employ it to improve association and cooperation around class material or to build integral educational experience to substitute or supplement in-person teaching, and others utilized it to make educational contents to support personalized education or academic cycles to combine self-study with cooperative educational material-driven education. Social media has increasingly turned into a central facet in promoting professional development (PD) and emerging professional identity (PI) for teachers (Kimmons and Veletsianos, 2014). Social media assisted as a method to nurture learning through the comprehension of others’ knowledge within a social community of peers (Sullivan et al., 2018). Teachers and educators have been using social media as a virtual learning tool both formally and informally for many years (Dutta, 2010). Indeed, social media along with its options have been found to pave the way for sustainable personal development and lifelong learning, which can be extended to all stages of life. Social media and relevant applications such as Ding talk, WeChat, and Tencent, are used as a platform that channels social capital, facilitating collaborations among people. These technologies can also be used to share teachers’ ideas and resources in the context of L2 teaching and learning; that is, teachers in various regions can share their ideas regardless of geographical limitations.

In addition, teaching approaches and strategies have been growing with the incorporation of technology specifically social media, which is maintained to be the pattern for future EFL academics (Xiao et al., 2020; Huang, 2021). It is noteworthy that during the occurrences brought about by the COVID-19 pandemic in 2020; teaching combined with ICT instruments is included in all colleges in expanding the classroom beyond the conventional classes. Nonetheless, educators might have a hard time carrying out online teaching due to the contrast of basic associations with in-person classrooms and the absence of knowledge and abilities preparation (Kohnke and Moorhouse, 2020). Language teachers need professional knowledge such as knowledge of language employed in syllabus perspectives, knowledge of language learning principles, knowledge of educational and evaluation and skills in the administration of the social facets of working with students, and such professional knowledge requires updating issues (Meng and Tajaroensuk, 2013). According to Gaytan and McEwen (2010), these attempts are mainly aimed at enabling teachers to effectively incorporate instructional social media into their teaching activities. Therefore, EFL teachers need to acquire the ability to create active learning settings where learning is enhanced. Given the emphasis put by countries on the knowledge economy in the twenty first century, which attaches enormous importance to creativity and change, some new challenges emerge regarding the difficulties faced by educators in their dealing with social media, as well as their PD (Mâţă, 2014; Yurtseven Avci et al., 2020). Wherein this aids educators to achieve a primary degree of usage, they desire to take part in training based on the curriculum that allows them to merge social media into their current curriculum (Luo et al., 2020). Yet, given some issues including time and cost, as pointed out by Kiedrowski et al. (2015), PD programs currently offered to teachers are far inadequate, particularly, concerning the use of social media. Some investigations have confirmed the necessity of providing additional PD for teachers regarding how to effectively apply emerging social media in their classroom (Kiedrowski et al., 2015).

Indeed, researchers consider PD as a pillar of educational reforms, which plays a pivotal role in enhancing learners’ achievement and engagement (Wei et al., 2010). PD goes a long way to enhance and develop teaching practices in the content areas, helping the teachers to gain a better understanding of assessment following standards, and using new tools and strategies in an innovative manner (Lawless and Pellegrino, 2007). PD brings about new information and opportunities to teachers thereby they embark on integrating technology into the classroom and can grasp how social media is associated with the curriculum (Tweed, 2013; Alberth et al., 2018).

Accordingly, research should focus on different dimensions of teacher education, along with the concepts associated with teaching (e.g., teacher cognition and teacher knowledge). Teachers’ PI or how EFL teachers view their professional roles plays an important role in making teaching effective (Pishghadam et al., 2019; Coombe, 2020). The notion of identity has been the focus of many disciplines and subdisciplines, including social psychology, history, and sociolinguistics, among others. Indeed, identity enjoys a special status in L2 education (Preece, 2016). Indeed, teaching is among the careers in which identity has an important function, and educators can learn how to employ expertise to build the domain of their PI by acting to affect the whole school (Beijaard et al., 2004). EFL Teachers’ identity comes into play when they are pushed to adopt new teaching practices. Such uncertainty may lead to perceived risk and lower teaching efficacy, which can bring about reduced motivation. This may induce a negative mentality and experience regarding using social media on the part of the teacher (Van Veen et al., 2005). In recent years, EFL educators and scholars have attached enormous importance to teachers’ PI and have emphasized its role in teacher education that is described regarding a specific complexity of one’s features assumed in contrast to that of different people (Pennington and Richards, 2016). That is, PI is a social construct that is manifested in the form of increased motivation, satisfaction with one’s job, perceived self-efficacy, and loyalty to one’s occupation, among others (Canrinus et al., 2012). PI plays a pivotal role in how EFL teachers make sense of their behaviors, and in how to act and grasp their work. Therefore, it might impact how teachers perceive the integration of social media in their teaching activities, as well (Seaman and Tinti-Kane, 2013; Badia and Iglesias, 2019; Liu et al., 2020). Indeed, educators’ PI refers to a central component of educators’ professional lives, which has an extensive influence on an educators’ education, PD, and so on, which affects personal education powers by affecting their real behavior within the education process (Derakhshan, 2022). A significant issue consistent with educators’ identity is commitment, which alludes to the fact that educational levels have gained significant vital consideration. Managing the educational section requires a higher educator’s professional commitment, defined as an affiliation, relation, and settlement of the place of work and liabilities to perform the assignments and tasks (Zhang et al., 2021). Zhang et al. (2021) argued that EFL teachers who had a high level of commitment to teaching have been found to integrate social media tools more willingly. Simultaneously, partaking in PD activities can help to improve commitment by assisting educators to develop their abilities and knowledge (Smith, 2005). Consistent with Park (2007), teachers with a great level of commitment to their job deal with high job fulfillment and identify themselves with the work of being an educator.

The application of social media to enhance personal development is in keeping with the current learning theory (e.g., Social Constructivism) (Felix, 2005). Following Social Constructivist Theory, learning is viewed as a social activity. This also applies to teachers, as well. Traditionally, teaching underestimated the collaboration among teachers, paying no attention to the important role of ideas and experience sharing among teachers. In recent years, social media has emerged as a tool to facilitate social interaction among learners and teachers. Using such technology, they can also share their teaching experience and resources, which plays a critical role in the personal development of teachers (Holmes et al., 2013). Although teachers are expected to take part in social media-focused PD tasks, they are often provided with no opportunity to select and plan the related activities as they are not trained in this domain. As a result, there may be no close ties between tasks and classroom practice, which results in a lack of commitment. Accordingly, the enhancement of PD plays a vital role in alleviating this problem, with multiple factors including teacher commitment and identity. A review of the literature shows an increasing recognition of the positive outcomes and benefits emanating from the social media-oriented programs for teachers’ PD. Some of these benefits include a notable decrease in teacher isolation and established PI (McLoughlin and Lee, 2010). Therefore, the study of factors such as teacher commitment and identity are deemed highly important in this regard for the reason that these factors assist the student teachers with learning how to teach and what it means to be a teacher (White and Lemieux, 2015). Recently, there has been an emergent concern in studying the PI of EFL teachers (Gu and Lai, 2019; Nguyen and Dao, 2019; Widodo et al., 2020). Regardless of the significance of social media in supporting PD (Heidari et al., 2020; Mart and Campbell-Barr, 2020), little is acknowledged about how a social media platform cares about English teachers’ PI. Indeed, based on the researcher’s knowledge, no studies have been done so far to take the role of both constructs, namely teachers’ PI and commitment in social media-focused PD since the significance of the issues includes facilitating teaching through informal learning settings, such as social media and beyond technology focused domain.

## Review of the literature

### Teachers’ professional identity

As for the instructional contexts, identity is described by Pennington (2015) as an impression and mental picture of what “being a teacher” means. This internal model determines teachers’ performance as they seek to put to practice such a mental model of being a teacher through specific “acts of teacher identity (Derakhshan and Nazari, 2022). L2 teachers take on several identities as they change their roles in social interaction. They come into contact with many players during this engagement (e.g., parents, students, colleagues, managers, public community, etc.). These roles are also influenced by arrangements, places, and objects in classrooms and institutions (Barkhuizen, 2016). Description of PI includes a relatively steady collection of features, views, values, incentives, experiences, and connections allowing people to describe their expert function (Dobrow and Higgins, 2005). As a dynamic concept, PI influences how a teacher behaves in the classroom. It also determines the efficacy of their teaching, as well as their perception of wellbeing. Moreover, PI impacts teachers” PD, enabling them to cope with educational changes. This, in turn, contributes to their motivation and creativity in their teaching (Abednia, 2012). Forming educators’ PI is a procedure of narrating and associating several I-positions and it is shaped in the path of self-participation as well as self-involvement and within the route of attempting to preserve consistency and continuity (Akkerman and Meijer, 2011). Currently, a large number of researchers have studied the notion of EFL educator identity (e.g., Mora et al., 2014; Ghanizadeh and Ostad, 2016; Labbaf et al., 2019). PI of educators includes inner and outer elements like educators’ individual life experiences, viewpoint on teaching, individual values and anticipations for the future, and educators’ connection with colleagues, career setting redesign, institutional framework, and the execution of teaching, academic improvement, school leadership, and PD (Kao and Lin, 2015).

The following roles have been identified by Beijaard et al. (2000) as three important aspects of PI: (a) teachers serving as professionals who have expertise in the subject matter. (b) Teachers serving as professionals who enjoy didactical expertise. (c) Teachers serving as pedagogical professionals. Indeed, teachers’ PI is a multifaceted, complicated, and changing procedure, during which several elements affect whether or not beginner educators pick to be passive or active (Kayi-Aydar, 2019). As teachers absorb identity positions, they might be discouraged and encouraged between their demands and selections and the cultural aspects in their setting (Dikilitas and Yayli, 2018). Zare-ee and Ghasedi (2014) presented the notion that educator PI is the way educators characterize their expert functions and combine them with individual functions influenced by elements inside and outside the class. Educator PI is developed based on how educators explain their career and how it is associated with other dimensions of their lives. Nevertheless, it has been decided that function and identity, i.e., practice and the center of teaching, are intertwined and frame educator growth together (Walkington, 2005). Teacher PI is mainly a functioning identity, which involves educators’ discernment of the various dimensions of educators’ function in the teaching career and is how educators view their career, give meaning to it, and play a role in it (Brenner et al., 2018). Hapsari et al. (2022) have done a study to investigate the role of LinkedIn-mediated activity shapes pre-service English teachers’ PI and to investigate the perceptions of pre-service English teachers on their participation in LinkedIn-mediated early PD activities. The findings revealed that LinkedIn-mediated early PD activities that raise awareness on developing the current professional digital footprint. According to Heidari et al. (2020), the use of online social networks in the context of higher education allows learners to enhance their professional identities by taking on the role of a mediator.

### Teacher commitment

Commitment is described as a driving force that shapes behaviors through constraining freedom and forcing people to follow a course of action in the face of conflicting reasons and demeanors (Meyer and Herscovitch, 2001). Commitment has to do with a demeanor or mental state that accounts for how a worker engages with his/her boss. This engagement eventually impacts whether or not he/she will be loyal to the institution (Meyer and Herscovitch, 2001). As for commitment in the school or university, one can say that scholastic commitment concerns the mutual associations between several elements, including psychological, relational, and surrounding (Human-Vogel, 2013). Commitment involves continuous engagement between several elements (e.g., individual, professional, and academic) (Choi and Tang, 2009). Given such a continuous interaction, some commitments take on more important roles than others in different circumstances. The outcomes resulting from various forces in one’s life determine the extent to which these commitments are strong (Choi and Tang, 2009). Teacher commitment refers to the extent to which he/she is willing to teach, playing an important role in teachers’ successful performance (Day, 2008) and such a type of commitment is necessary for effective instruction as it pushes the teachers to proceed with their profession. Teacher commitment is indicative of educators’ perceived loyalty to the organization where they work. Such a commitment has been found to predict learning and mental results (Day, 2008). Observations show that committed educators can contribute to creating an environment where learners are energetic and motivated by encouraging the learners to follow up on their exercises. Such a type of commitment is a requirement for effective teaching, as it involves being committed to students, the school, vocational success, proficient knowledge, and the teaching profession (Crosswell and Elliott, 2004). Engaging in interaction with students and paving the way for their growth are appealing to committed teachers. These teachers seek to promote their educational proficiency by using several methodologies. Consequently, education would fail if teachers lacked enthusiasm. Highly committed teachers find teaching very appealing (Choi and Tang, 2009). Teachers’ commitment to their teaching affords them both the chances of PD, helps them involve learners, and provides numerous tactics and learning models that outfit learners’ situations (Asiyah et al., 2021). Also, PD tasks have the prospective to serve as association-building activities that have a constructive effect on teachers’ commitment. Teachers’ commitment being an inner issue of educators’ performance indicated how their commitment was crucial to their presentation as there was a robust association between learning and teacher work commitment that is taken into consideration in PD activities (Asiyah et al., 2021).

### Professional development

As a context-specific phenomenon, PD is a continuous effort guided by standards, manifested inside the pedagogical activities, focuses on students’ learning, and is tailored to educators’ levels of career growth. The PD programs seek to enhance educational quality as it is an essential component of learning (Schlager and Fusco, 2003). PD is characterized as actions that have the objective of improving expert development (Goh and Loh, 2013), thereby assisting educators with comprehending teaching and educational cycles better and elevating their awareness of their students (Darling-Hammond and McLaughlin, 2011). PD can also be characterized as constantly building educators’ knowledge and expert abilities throughout their academic profession, during which professional educator identity is formed and theoretical knowledge is changed into practice (Kuijpers et al., 2010). The PD aims at increasing educators’ quality because it is a vital component of learning (Geringer, 2003). Engaged in learning, educators come to show cooperation, thoughtfulness, and eagerness for spotting and solving problems related to education and learning. Furthermore, the emphasis of PD is on the long-run development of educators and is a dynamic, practice-centric, and continuous cycle that combines teaching practices and PD actions (Hos and Topal, 2013). Moreover, PD has to do with educators’ learning, paving the way for them to put to use their knowledge for the sake of learners’ growth (Avalos, 2011). PD can play an important role in improving tasks. Undoubtedly, sticking to the goals set by the educators, as well as the local standards, or the learners’ evaluation (individual and collective) can contribute to enhancing the PD in the best way (Colbert et al., 2008). Thanks to PD, people internalize their knowledge and skills related to their job, which may surface in various shapes (e.g., formal educational classes to gaining experiential learning through daily activities); however, PD commonly refers to the enhancement of professional knowledge by engaging in short formal courses offered through working teams (Collin et al., 2012).

### Social media

The majority of social media encompass references to “internet-based” or “online” communication such as micro-blogs, blogs, and social network websites. The other significant component describing the term refers to sharing data employing “basically net or mobile-based apps” or, more generally, “formats of e-communicating” permitting it. The term is highly ambiguous, such that it is primarily employed nowadays to define roughly any sites on the internet,” considering that even conventional media may occasionally be taken into account social media as “the border drawn between both is gradually narrowing as both keep on evolving (Nations, 2017). Several reasons exist why social media is recommended to assist the occupational expansion of educators within intercontinental schools. In fact, they can be easily employed and are omnipresent, therefore, are needless of education that permits individuals to communicate with people with identical hobbies, tasks, history, or in-person relationships and offer a virtual environment for learning (Srivastava, 2012). Social media proposes a low-cost platform for the construction of a PI or personal brand, connecting who an individual is within and outside his corporation (Dutta, 2010). Booth (2012) believes that these learning settings offer an internet-based output to assist personalized, related, occupational learning and provide access to sources with no location or monetary constraints. In addition, using such instruments may help educators have lower feelings of isolation (Visser et al., 2014). Social media assimilated to other approaches that care about PD tasks for online capability, comprising explanation of formal prospects and personnel provision (Coswatte Mohr and Shelton, 2017). It may ease educators’ required source of exchange. Since educators select PD for new opinions and tactics following their material areas and class learning goals (McCray, 2018), searching for the resource is a major reason for educators’ employment of social media for PD (Hunter and Hall, 2018). Internet-based, educators can gain access to a large extent of sources; however, educators take into account social media as a confident resource for the majority of updated activities and tactics (Krutka and Carpenter, 2016; Trust et al., 2016). In other words, teachers develop professionally when they are afforded opportunities to engage as learners in collaborative settings, for example, through the collective construction of pedagogical knowledge, the creation of new instructional practices or the presentation of optimal solutions to classroom issues. All these activities, underpinned by reflective dialogue, can be facilitated by modern technology, and this is where blogging comes into play (Cirocki and Farrell, 2017). Secondly, social media may ease educators’ social construction. Outside the amount and quality of sources gained in social media, educators employ social media to satisfy their social and affective requirements as well.

Given the contribution of social media to the personal development of teachers, investigations have been carried out on the potential role played by social media in PD. As an example, Holmes et al. (2013) examined the effects of Twitter on teacher PD. The findings showed that the social networking site was a useful tool through which students can access both instructive educational resources and social support. The study reached the conclusion that Twitter can be used as a versatile resource for sustainable teacher PD. In the same vein, Jenkins et al. (2009) found out that Twitter makes it possible for pre-service teachers to keep in contact with practicing teachers by helping them to share their ideas and discussions. This helps the pre-service teachers to practice in the field; moreover, social networking site also allows teachers to keep track of their own PD (Risser, 2013). Qi and Wang (2018) proved the use of WeChat as a social media platform for language teachers’ PD. Carpenter (2015) conducted a study to explore the application of Twitter among preservice teachers. The findings revealed that most participants perceived the positive role of Twitter in education, expressing their desire to use the platform for their future professional purposes. In the same vein, Xue et al. (2021) examined the way some teachers in China resorted to WeChat to create an online Community of Practice for purpose of fostering their professional learning. To this end, they benefitted from mobile technology-enhanced teaching. The findings showed that engaging in the teacher group activities enhanced the teachers’ professional knowledge, leading to a noticeable improvement in their teaching practices.

### The interrelation among teachers’ professional identity, commitment, and social media-focused professional development

The social media-focused PD platform focuses on an extensive range of positioned, social, and distributed learning chances for language principals while distorting the lines between formal and informal PD. With the emergence of social media, the customary learning societies that were initially prearranged as offline formal PD inventiveness may develop into massively circulated online learning. This can nurture significant modifications in a principal’s education and learning performance (Gruzd et al., 2012). Taking part in social media-focused PD can help teachers as it triggers their commitment. Indeed, their commitment is nurtured to the extent to which the teaching occupation is delivered to teachers through social media and it brings about success in their PD and progress. Using social media for PD is a spontaneously accessible technological source that is universal and appropriate; therefore, teachers can commit in PD during times outside of arranged classroom teaching in a setting of their choice. PI is a central issue in teacher growth, attrition, and career gratification (Hong et al., 2018) and social media provide prospects for teachers to interact that can help their PI development (Carpenter, 2015). Teacher PI is a principal issue in present conceptualizations of PD as it assists to capture the way teachers cultivate modified considerations that regulate their involvement and also root the teachers in a diversity of socio-cultural and official discriminations (Beijaard, 2019). Undoubtedly, identity is so dominant to teacher PD that Beijaard (2019) insistently identified that “teacher learning, consequently, can and should be conceptualized as teacher identity learning” (p. 1). Reflecting on teachers about the identities they have when cooperating in a social media-focused context can be supportive of their commitment. Moreover, identity has been defined as the involvement in “borderland discourses” whereby a teacher will promote their practiced self by getting on their particular principles, classroom performance, and PD capabilities (Alsup, 2006).

## Conclusion

As a central component of any academic improvement, educators must be expertly well-prepared to carry out any improvement successfully and deal with present difficulties in teaching and educational cycles (Dayoub and Bashiruddin, 2012). Nonetheless, they must do it “innovatively” to assist in bringing about actual alterations in educator practice and enhancing learners’ success (Gulamhussein, 2013). As individuals increase their use of social media and the settings in which they live and work, they can create and frequently change their identity over time concerning the new people and collections that they cooperate with others and the new functions they accept in the situation of the action. The implementation of social media for PD is without a doubt a contemporary occurrence expedited by improvements in technology. Language teachers’ use of social media in teaching activities through PD tasks means that these teachers are highly dedicated to their profession, which shows they are intrinsically motivated to carry out any type of task they are asked for. These EFL teachers have even more motivation when they deal with performing demanding tasks since the challenging aspect of the task leads to the highest level of commitment and intrinsic motivation (Deci and Ryan, 2016). In this way, the use of social media-based PD activities enhances EFL teachers’ commitment by providing them with an opportunity to promote their skills and core content knowledge. It can be determined that PD is likely to enhance teacher commitment by strengthening their perception of efficacy, improving their motivation and freshness, and satisfying their perceived need for growth (Hausman and Goldring, 2001).

Besides, educators’ PI impacts the way they understand and respond to information during their learning and when participating in educator readiness programs and while taking part in PD in the process of teaching (Luehmann and Tinelli, 2008). Moreover, thinking about educators’ PI provides language teachers a beneficial lens into ways of education and how they build and rebuild their perspectives of their roles as language educators regarding their mates and the setting that it helps them to actively get involved in the preparation of content, trying to incorporate what they learn into their teaching that can also reinforce EFL teachers’ commitment to teaching. Viewed from this perspective, teacher PD contributes to teacher professionalism, which could impact their commitment positively and can also increase the likelihood of their retention in school and their profession.

It can be concluded that the social media-focused PD contributes to the beneficial and positive development of EFL teachers’ PI since the social media-focused PD programs increase the EFL teachers’ motivation for integrating what they gain from the programs into their teaching. Undeniably, teachers establish the imagined identity through engaging in social media-focused activities that in turn, result in the establishment of a new identity. EFL teachers’ involvement, interaction, and negotiation in social media-focused communities may push them to construct and reconstruct their PI. Because identity is a variable construct, educators’ PI is built and expanded step by step due to participation in a social media-based society which provides them a basis for socializing and cooperating with other educators to nurture their PD that bring about success in the profession.

## Implications and suggestions for further research

Technology is regarded as an essential element of teaching and learning that has the capability to support learners’ achievement so enlightening technology integration through social media in language learning depends on the growth of robust, comprehensible PD programs that are planned with a strong comprehension of how EFL teachers employ social media. On the basis of this review, one can refer to the significance of social media as an instrument to be incorporated in PD programs while implementing it in the classes needs teachers’ commitment. Sufficient PD for EFL educators regarding the use of social media in their classroom can assist them to apply tactics that also enhance their success. Successfully using social media in classes happens merely while pre-service educators consider social media usage modeling within the overall preparation and gain possibilities to learn and employ social media in instructional environments that can be done through PD.

Moreover, this review has practical implications which allude to PD because educators attempt to ease learning by merging social media which requires educators’ PI and commitment for its development. As a result, educators’ consciousness of their commitments and identities can be taken into account as critical elements in PD, as it can be also useful in their overall improvement. It is crucial to enhance educator recognition of the important function of educators’ PI and commitment to their career and the elements that build it. Experienced educators must also be aware of the fundamental prompts of alteration in their PI and assist novice educators with having a good comprehension regarding it and its development from the beginning. Colleges need to equip educators with competencies to apply social media and also assist educators in converting how they consider education as a job and how an educator should be practically educated. PD programs are required to encompass different choices wherein educators are provided with the opportunity to experience learning from some instruments and tactics simultaneously, to completely experience the limitations and affordances of each of them. Beyond learning how to employ specific digital tools, assets, and social media, teacher training databases, colleges and policymakers must arrange for suitable care to the educators through their identity development as their PI has a noteworthy effect on their confidence, presentation, in addition to their educational selections.

The present research can also be used by educator trainers engaged in programs of pre-service and in-service educator instruction. They can specify educators’ weak points and powers from their perspective regarding the degree of commitment and educators’ PI by holding debate forums to provide them with several classes and programs. Indeed, based on the research review, EFL teachers’ commitment to the use of social media in L2 pedagogy increases through their engagement in the social media-focused teacher preparation programs. Moreover, teacher educators can provide teachers with numerous PD opportunities to better understand and experience the use of social media. Technology integration through social media in this domain can be useful for both teachers, who should use such tools in the classroom, and for teacher educators, who do well to make use of social media to draw on teachers’ previous knowledge and strengths. By employing social media, educators may cooperate and exchange opinions regarding educational activities with both local educators with identical expertise, and with those from outspread academic professionals from various sections of the world (Trust, 2012). Using social media, educators can react to opinions at a suitable time. This social and expert interplay and exchanging comments assists educators think about their educational activity for exposure to new opinions and viewpoints, therefore enhancing their educational activity. Educators employing the technologies of social media to improve their own technical information and expert growth can better teach learners to efficiently and ethically employ them (Trust, 2012).

It can also be used by syllabus designers, who are able to gain a new understanding of how teachers come to view the role of such tools in their teaching activities. Moreover, EFL teachers should be helped in their attempts to lay out a lesson plan that merges such equipment and tactics into their particular material, and this assistance should be consistent as they apply the instruments and tactics practically with learners. Academic supervisors, managers, and those accountable for the TPD growth in schools have to understand the possibility of internet-based expert growth nets to increase conventional types of educator PD. Presenting workshops on the significance of active learning totally *via* social media tools slightly allows educators to enjoy how a learner-oriented task can make contributions to learning or provide them with the experience of selecting and applying the suitable tactic or instrument for their class.

Considering the multifaceted and hierarchical nature of higher education educators’ PI, future empirical studies may be carried out to search dimensions of PI that synergistically work with educators’ learning social media integration; how PI is changed through time in the social media setting; and how colleges can ease the PI rebuilding procedure. Qualitative studies can be conducted in this domain through the interview as the results of the interview can show the identity and its effect on educators and it can reveal how it feels to be an educator in academic environments nowadays and resolve arguments between the individuals and the setting where they live. The current investigation into teacher identities is a vigorous instrument for encouraging a superior indulgence of the teaching profession and their roles in diverse circumstances and times. Additional research should be done to explore how educators’ experience in implementing social media might form their forthcoming education and identities.

## Author contributions

The author confirms being the sole contributor of this work and has approved it for publication.
